# Telomerase therapy attenuates cardiotoxic effects of doxorubicin

**DOI:** 10.1016/j.ymthe.2020.12.035

**Published:** 2021-01-01

**Authors:** Shambhabi Chatterjee, Teresa Hofer, Alessia Costa, Dongchao Lu, Sandor Batkai, Shashi Kumar Gupta, Emiliano Bolesani, Robert Zweigerdt, Diego Megias, Katrin Streckfuss-Bömeke, Christina Brandenberger, Thomas Thum, Christian Bär

**Affiliations:** 1 Institute of Molecular and Translational Therapeutic Strategies (IMTTS), Hannover Medical School, Hannover 30625, Germany; 2 REBIRTH Center for Translational Regenerative Medicine, Hannover Medical School, Hannover 30625, Germany; 3 Leibniz Research Laboratories for Biotechnology and Artificial Organs (LEBAO), Department of Cardiac, Thoracic, Transplantation, and Vascular Surgery, Hannover Medical School, Hannover 30625, Germany; 4 Confocal Microscopy Unit, Spanish National Cancer Centre (CNIO), Madrid 28029, Spain; 5 Clinic for Cardiology and Pneumology, University Medical Center, Göttingen 37075, Germany; 6 DZHK (German Center for Cardiovascular Research), partner site Göttingen, Göttingen 37075, Germany; 7 Institute of Functional and Applied Anatomy, Hannover Medical School, Hanover, Germany; 8 Biomedical Research in Endstage and Obstructive Lung Disease Hannover (BREATH), Member of the German Center for Lung Research (DZL), Hannover 30625, Germany; 9 Fraunhofer Institute for Toxicology and Experimental Medicine, Hannover 30625, Germany

**Keywords:** telomerase, doxorubicin cardiotoxicity, mitochondria, AAV gene therapy, heart failure, cancer, anthracyclin, telomere, ROS, cardio-oncology

## Abstract

Doxorubicin is one of the most potent chemotherapeutic agents. However, its clinical use is restricted due to the severe risk of cardiotoxicity, partially attributed to elevated production of reactive oxygen species (ROS). Telomerase canonically maintains telomeres during cell division but is silenced in adult hearts. In non-dividing cells such as cardiomyocytes, telomerase confers pro-survival traits, likely owing to the detoxification of ROS. Therefore, we hypothesized that pharmacological overexpression of telomerase may be used as a therapeutic strategy for the prevention of doxorubicin-induced cardiotoxicity. We used adeno-associated virus (AAV)-mediated gene therapy for long-term expression of telomerase in *in vitro* and *in vivo* models of doxorubicin-induced cardiotoxicity. Overexpression of telomerase protected the heart from doxorubicin-mediated apoptosis and rescued cardiac function, which was accompanied by preserved cardiomyocyte size. At the mechanistic level, we observed altered mitochondrial morphology and dynamics in response to telomerase expression. Complementary *in vitro* experiments confirmed the anti-apoptotic effects of telomerase overexpression in human induced pluripotent stem cell-derived cardiomyocytes after doxorubicin treatment. Strikingly, elevated levels of telomerase translocated to the mitochondria upon doxorubicin treatment, which helped to maintain mitochondrial function. Thus, telomerase gene therapy could be a novel preventive strategy for cardiotoxicity by chemotherapy agents such as the anthracyclines.

## Graphical abstract

**Figure:**
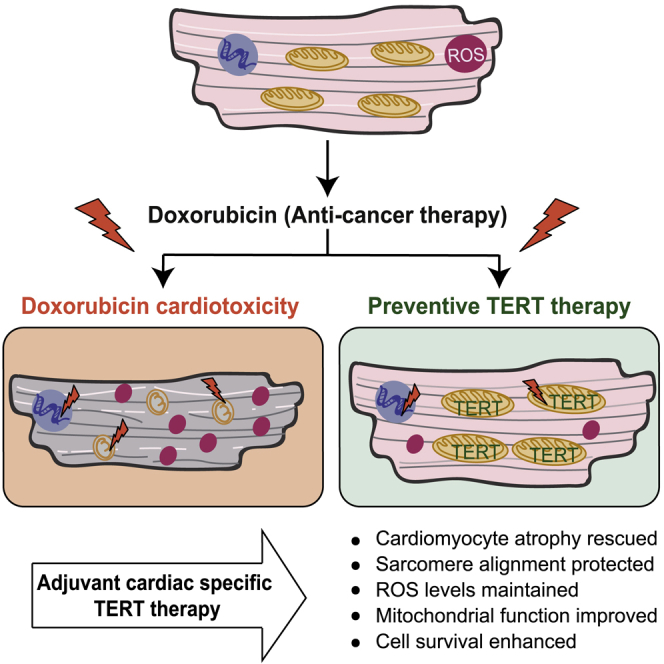

## Teaser

Bär and colleagues applied a telomerase gene therapy to prevent cardiotoxicity upon doxorubicin anti-cancer treatment leading to enhanced cardiomyocyte survival in a DOXTOX mouse model and human cardiomyocytes. Cardioprotection is at least partially mediated through non-canonical functions of telomerase, which translocated into mitochondria upon doxorubicin stress to maintain mitochondrial function.

## Introduction

Significant advances in modern medicine have led to a strong increase in survival of cancer patients. However, the improved anti-cancer therapeutics also led to major health concerns among the cancer survivors. Apart from reoccurrence of tumors, toxic effects of anti-cancer medication on the heart frequently lead to temporary dysfunction or severe long-term cardiovascular diseases (CVDs), including myocardial toxicity, hypertension, and arrhythmias, which can ultimately result in heart failure.^1^ CVDs emerged as one of the most life-threatening outcomes of anti-cancer drugs, which severely impair the lifestyle of cancer survivors. The severity of treatment-induced side effects depends on several factors, such as duration and type of anti-cancer therapy and existing comorbidities. The precise molecular reasons why some patients are more prone than others to develop cardiotoxicity are largely unknown. The incidence of heart failure among cancer survivors has been investigated in several large patient cohorts, and depending on the cancer malignancy and therapy provided, up to 11% of patients succumbed to CVDs and heart failure.^2^^,^^3^ One of the contributing factors for heart failure is the inability of adult hearts to regenerate cardiomyocytes (CMs) that are lost, directly or indirectly, in response to anti-cancer treatments. In contrast, lower vertebrates (e.g., amphibians and fish), neonatal mice,^4^ and potentially newborn humans^5^ can fully regenerate the heart after an insult, which is mediated via cardiomyocyte proliferation. This regenerative potential correlates with high cardiac telomerase expression but is rapidly lost after birth, coincidental with the silencing of telomerase.^4^^,^^6^ Interestingly, overexpression of telomerase in the adult mouse heart via AAV9 gene therapy was shown to confer cardioprotection after myocardial infarction.^6^ The enhanced survival was accompanied by a modest increase in cardiomyocyte proliferation. Nonetheless, the extent of cardiomyocyte regeneration induced after the TERT gene therapy was inadequate to explain the level of improvement in cardiac function. Altogether, this suggests a crucial role of telomerase in cardiac survival processes, but indeed the molecular mechanisms underlying telomerase-mediated cardioprotection remain ill-understood.

Telomerase is a ribonucleoprotein complex that maintains telomeres by *de novo* addition of telomeric DNA repeats (5′-TTAGGG-3′) to the ends of chromosomes.^7^ Besides this canonical function, the catalytic subunit TERT (telomerase reverse transcriptase) was shown to shuttle between the nucleus and mitochondria, where it is involved in the detoxification of mitochondrial reactive oxygen species (ROS). This has first been demonstrated in a fibroblast cell line^8^ and recently also in blood vessels from patients with coronary artery disease (CAD).^9^ The effects are *TERT* specific, since pharmacological activation or inhibition of *TERT* resulted in protection from ROS-provoked apoptosis or led to higher levels of mitochondrial ROS, respectively.^9^^,^^10^ This might be extremely relevant for CMs due to their high mitochondrial density, which renders them particularly vulnerable to ROS, resulting in apoptotic signaling and essentially CM death.^11^

This led us to ask the question whether CM-specific *TERT* expression can ameliorate the cardiotoxic consequence of chemotherapeutic agents, which are at least partially attributable to increased ROS production in CMs.^3^ Doxorubicin is one of the most effective anthracycline drugs, which can act against a wide range of cancers. However, its clinical application is severely limited because of potential short- as well as long-term cardiotoxic effects.^3^ Thus, we investigated whether CM-specific *TERT* overexpression might be particularly effective against doxorubicin-induced toxicity. To this end, we reconstituted *Tert* expression in a mouse model of chronic doxorubicin-induced cardiomyopathy followed by overexpression of *hTERT* in a translational platform comprising human-induced pluripotent stem cell-derived CMs (hiPSC-CMs) to gain further insights into the cardioprotective roles of telomerase.

## Results

### *Tert* protects from doxorubicin cardiotoxicity *in vivo*

**Figure 1:**
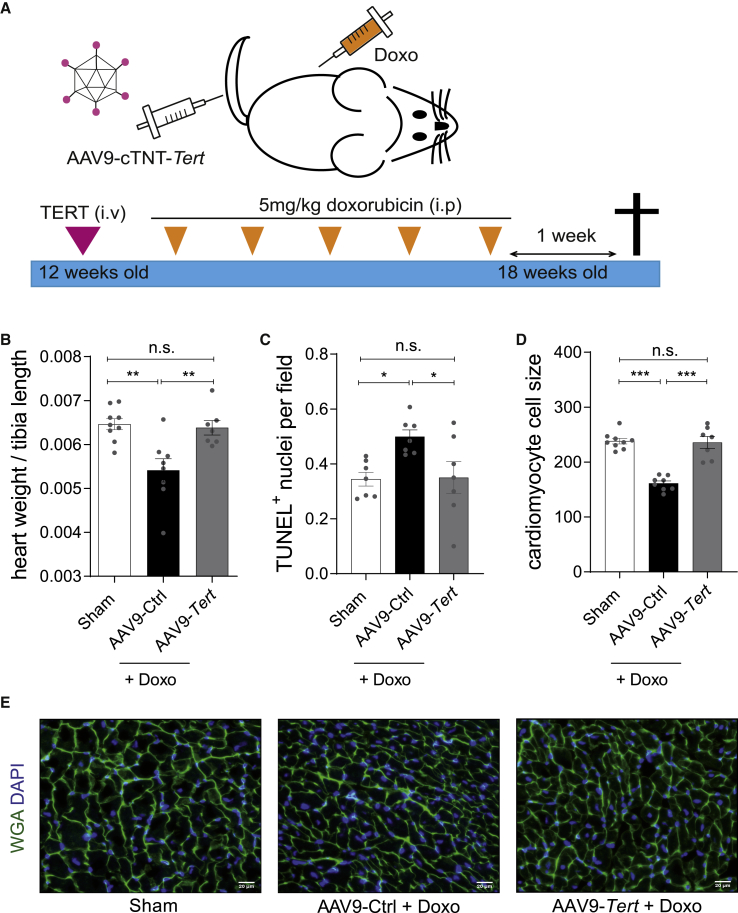
AAV9-*Tert* prevents doxorubicin-induced cardiac apoptosis and atrophy (A) The mouse model of doxorubicin-induced toxicity used to study the therapeutic effects of telomerase overexpression *in vivo*. Mice (12 weeks) were injected with AAV9-*Tert* (intravenously), and 5 mg/kg doxorubicin (intraperitoneally) was administered for 5 consecutive weeks. One week later, the animals were euthanized, and tissue samples were harvested for further analysis. (B) Decline in heart weight upon doxorubicin administration was preserved in mice injected with AAV9-*Tert* (n = 9/8/7 mice). (C) The AAV9-*Tert* therapy rescued cell death as measured using TUNEL assay (n = 7 mice in each group). (D) The AAV9-*Tert* therapy rescued CM cell size (n = 9/8/7 mice). The average cell size from each animal (n = 30 cells) has been plotted. (E) Representative images of WGA staining of transverse heart sections used to measure cell size. AAV9 particles were injected at a dose of 1 × 10^12^ vg/mouse. Doxo, doxorubicin. ∗p < 0.05; ∗∗p < 0.01; ∗∗∗p < 0.001; n.s., not significant; one-way ANOVA, Tukey multiple-comparisons test.

To test putative beneficial effects of *Tert in vivo*, we employed a mouse model of chronic doxorubicin-induced cardiotoxicity. Mice (12 weeks) were injected with cardiotropic AAV9 particles (1 × 10^12^ vg/mouse) to express *Tert* under the control of the CM-specific cardiac troponin T (cTNT) promoter. After 1 week to allow full *Tert* induction in the heart, mice were injected weekly for 5 consecutive weeks with doxorubicin (cumulative dose of 25 mg/kg body weight [BW]) and were sacrificed 1 week later (Figure 1A). Stable *Tert* expression in the heart throughout the experiment was confirmed by quantitative real-time PCR (Figure S1A). As expected, the chemotherapy treatment caused significant weight loss irrespective of the treatment with AAV9-*Tert* or AAV9-empty control (AAV9-Ctrl) virus (Figure S1B). Surprisingly, the reduction in heart weight/tibia length ratio in doxorubicin-treated control mice was completely rescued in the *Tert-*treated group (Figure 1B). Doxorubicin treatment significantly increased cardiac apoptosis, as measured by the number of TUNEL-positive cells in AAV9-Ctrl mice, whereas the level of apoptosis in mice with *Tert* therapy was comparable to the sham group (Figure 1C). Since doxorubicin stress leads to cardiac atrophy,^12^^,^^13^ we tested whether *Tert* can also rescue this pathological effect. Indeed, mice treated with doxorubicin showed significant cardiomyocyte atrophy, whereas AAV9-*Tert* gene therapy completely rescued this effect (Figures 1D and 1E). Thus, our results suggest that CM-specific *Tert* expression preserves the cardiac structure by preventing apoptosis and atrophy in CMs.

### *Tert* protects from doxorubicin-induced cardiac dysfunction

**Figure 2:**
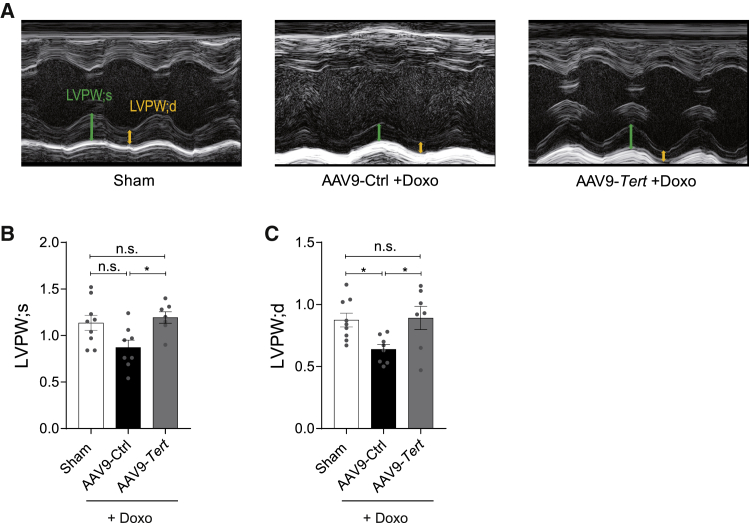
AAV9-mediated *Tert* overexpression rescues cardiac atrophy in doxorubicin-induced cardiotoxicity model (A) M-mode echocardiography of mice injected with *Tert* and then treated with saline (Sham) or injected with AAV9-*Tert* or control virus followed by doxorubicin treatment. Arrows indicate systole (green) and diastole (yellow) and highlight the difference. (B and C) Left ventricular posterior wall at end-systole and -diastole was assessed by echocardiography (n = 9/8/7 mice). AAV9 particles were injected at a dose of 1 × 10^12^ vg/mouse. LVPW, left ventricular posterior wall at end-systole (s) or -diastole (d). ∗p < 0.05; one-way ANOVA, Tukey multiple-comparisons test.

**Figure 3:**
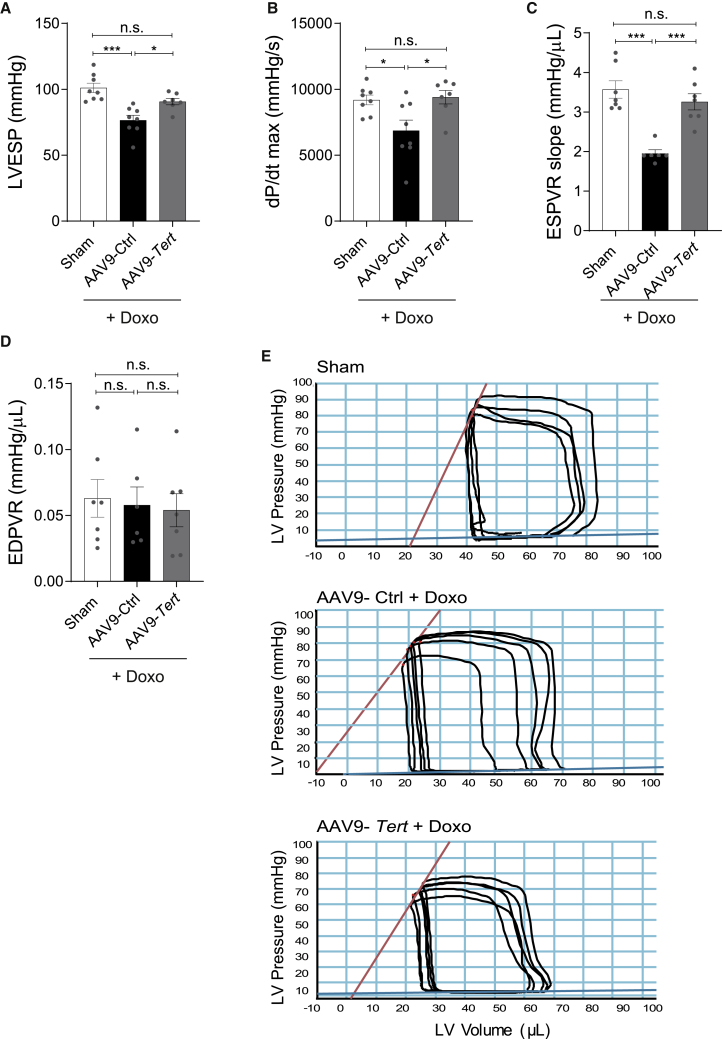
Effect of AAV9-mediated *Tert* overexpression on doxorubicin-induced systolic dysfunction (A–E) Measures of global systolic function (LVESP and dP/dT) (n = 8/8/7 mice) (A and B); load-independent systolic and diastolic parameters (ESPVR, EDPVR) (n = 7/6/7 mice) (C and D); and representative pressure-volume (PV) loops (E) obtained with a PV conductance catheter system at varying preload using transient vena cava occlusion, showing differences between mice injected with AAV9-*Tert* or control virus and then treated with saline or doxorubicin. AAV9 particles were injected at a dose of 1 × 10^12^ vg/mouse. LVESP, left ventricular end-systolic pressure; dP/dt max, ventricular contractility assessment; ESPVR, end-systolic pressure volume relationship; EDPVR, end-diastolic pressure volume relationship. ∗p < 0.05; ∗∗p < 0.01; ∗∗∗p < 0.001; one-way ANOVA, Tukey multiple-comparisons test.

Prior to sacrificing, all mice were subjected to cardiac function analyses by means of echocardiography and invasive hemodynamic measurements. We confirmed doxorubicin’s effect of reduced heart weight (HW), cardiac volume, and function^13^, ^14^, ^15^ (Tables S1 and S2). Overexpression of *Tert* also exhibited beneficial effects on the systolic and diastolic cardiac dimensions, as observed in M-mode echocardiography. The left ventricular (LV) posterior wall dimensions (LVPW systole [LVPW;s] and LVPW diastole [LVPW;d]) were significantly reduced after treatment with doxorubicin but were rescued in the *Tert* group, further corroborating the rescue from cardiac atrophy (Figures 2A–2C; Table S1). In line with the enhanced HW/BW ratio, LV mass was increased in the *Tert* group, although without reaching statistical significance. On the functional level, doxorubicin treatment caused a significant reduction in LV end-systolic pressure (LVESP) (Figure 3A). Moreover, as a measure of global LV contractility, the maximum first derivative of ventricular pressure with respect to time (dP/dt max) was also decreased (Figure 3B). Importantly, both parameters were significantly improved in *Tert*-treated mice. When we further investigated the systolic cardiac function by measuring the end-systolic pressure-volume (PV) relationship (ESPVR), we found that this load-independent index deteriorates under doxorubicin stress conditions, indicating decreased contractile function. *Tert* overexpression improved this parameter (Figures 3C and 3E). Global diastolic parameters and the end-diastolic PV relationship (EDPVR) were not altered in our doxorubicin stress model (Figure 3D; Table S2). Taken together, our data demonstrate that *Tert* re-activation prevents the functional decline of the heart caused by doxorubicin chemotherapy.

### Protective effects are mediated through non-canonical TERT function

**Figure 4:**
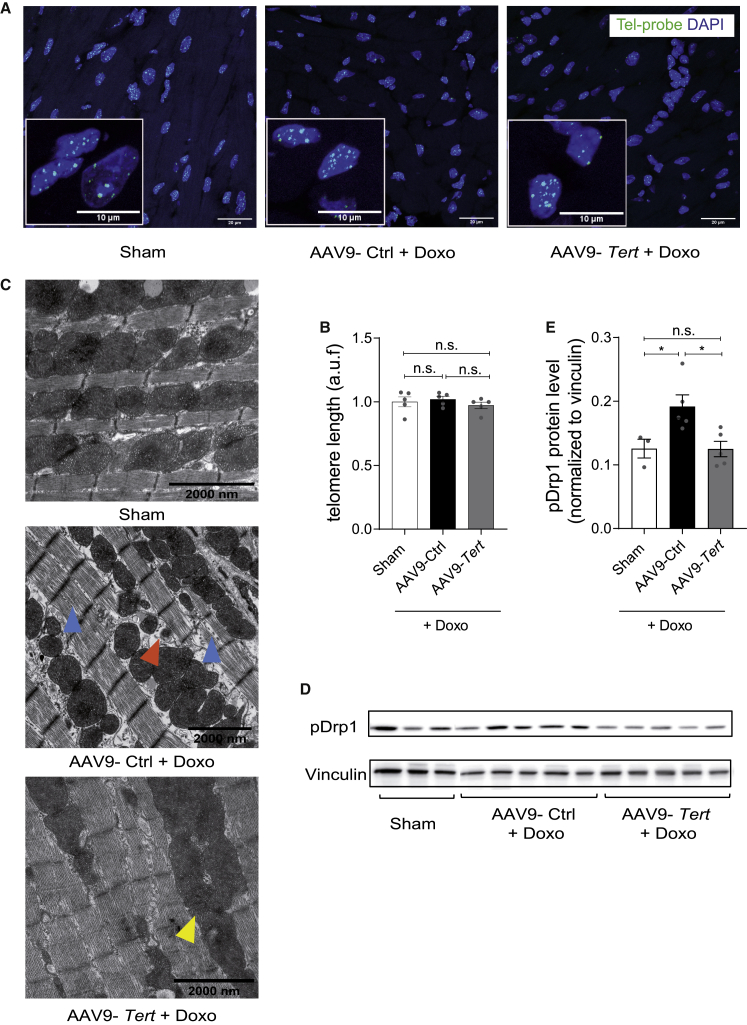
AAV9-mediated *Tert* cardioprotection is conferred by non-canonical telomerase functions at the mitochondria (A and B) The telomere length in mouse heart does not increase upon overexpression of *Tert* as measured by qFISH (n = 5 mice/group). Scale bars indicate 20 μm, and, for insets, 10 μm. (C) Ultra-structure analysis using transmission electron microscopy indicated that the mitochondria undergo more fission post doxorubicin treatment (orange arrowhead), which was rescued partially by telomerase therapy (yellow arrowhead). Blue arrowhead indicates disrupted myofibril alignment. Scale bars indicate 2 μm. (D and E) Western blot depicting the levels of phosphorylated Drp1 (pDrp1), mitochondrial fission protein (n = 3/5/5 mice/group). AAV9 particles were injected at a dose of 1 × 10^12^ vg/mouse. a.u.f = arbitrary unit of fluorescence. ∗p < 0.05; one-way ANOVA, Tukey multiple-comparisons test.

The canonical role of telomerase is to add and maintain hexameric telomere repeats at the ends of the eukaryotic chromosomes.^16^ To gain insight into the molecular mechanism underlying TERT-mediated cardioprotection, we first measured the telomere length in heart tissue. We did not observe any differences in telomere lengths in response to doxorubicin, irrespective of treatment with AAV9-Ctrl or AAV9-*Tert* particles (Figures 4A and 4B). Since doxorubicin is known to elevate ROS production, we measured the levels of mitochondrial superoxide dismutase (SOD2) and catalase.^17^ While SOD2 was unchanged, we found significantly lower levels of catalase in *Tert*-treated mice compared with the AAV9-Ctrl group, suggesting that *Tert* helps to maintain lower levels of ROS (Figures S2A and S2B). Furthermore, we analyzed heart tissue morphology using transmission electron microscopy and observed severe misalignment of the sarcomeres upon doxorubicin treatment (Figure 4C, blue arrows). The disrupted myofibrils were also accompanied by fragmented mitochondria in the mice administered with doxorubicin (Figure 4C). Strikingly, the telomerase overexpression was able to rescue the sarcomere organization. The relative frequency of mitochondrial fission and fusion events maintain healthy mitochondrial dynamics. Doxorubicin is known to induce high levels of mitochondrial fission, leading to mitochondrial damage.^18^ Notably, the mitochondria appeared to be more fused to each other in AAV9-*Tert*-treated mice (Figure 4C, yellow arrows), which was in line with lower levels of the protein phospho-Drp1, indicating toward less mitochondrial fission (Figures 4D and 4E).

### *TERT* is silenced in mature hiPSC-derived cardiomyocytes

**Figure 5:**
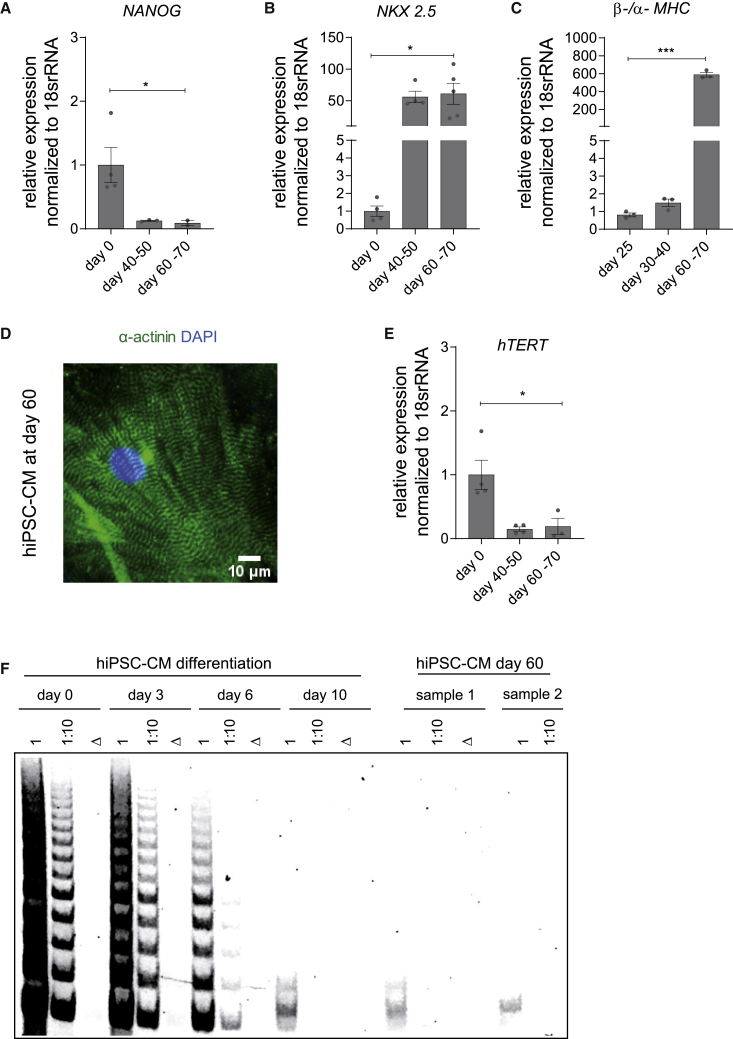
Establishment of hiPSC-CMs as a translational research platform for AAV6-mediated *hTERT* overexpression (A–C) Expression of pluripotency marker (*NANOG*) (n = 4/3/2 independent differentiation experiments) (A), cardiac-specific lineage marker (*NKX2.5*) (n = 4/4/5 independent differentiation experiments) (B), and ratio of myosin heavy-chain isoforms (*β-/α-MHC*) (n = 3 independent differentiation experiments) (C) during differentiation and prolonged cultivation of hiPSC-CMs. (D) Purified hiPSC-CMs at day 60 exhibited well-organized sarcomere structures as seen by α-actinin immunostaining. (E) The mRNA expression of *hTERT* during CM differentiation protocol indicated downregulation by day 60 (n = 4/4/3 independent differentiation experiments). (F) Telomerase activity from cell lysates collected at various time points of the differentiation timeline showed a gradual decrease in TRAP activity by day 10 and undetectable by day 60. Δ indicates heat-inactivated cell lysate; 1 indicates undiluted cell lysate; 1:10 indicates cell lysate diluted by factor 10; scale bar indicates 10 μm. All data are mean fold change relative to control ± SEM. ∗p < 0.05; ∗∗∗p < 0.001; two-tailed unpaired t test with Welch’s correction.

To further investigate the *hTERT* therapy in a translational platform, we sought to apply the doxorubicin-induced cardiotoxicity model in hiPSC-CMs. The unlimited production of human cardiomyocyte-like cells makes the hiPSC-CM model a unique and desirable tool for *in vitro* translational experiments. Since most human tissues, including the heart, are devoid of detectable *hTERT* expression, we first determined whether *hTERT* is silenced during the differentiation of human embryonic stem cells (hESCs) and hiPSCs into CMs and whether such CMs may serve as a model to study potential cardioprotective effects of *hTERT* as observed in rodents.^7^^,^^8^ The expression of *hTERT* mRNA was measured every day through a defined 10-day differentiation protocol. As expected, *hTERT* was readily detectable in pluripotent cells but gradually decreased during the differentiation process (Figure S3A). Cardiac lineage specification was confirmed by the loss of pluripotency markers (*NANOG*) (Figure 5A) and simultaneous emergence of CM-specific gene expression (*TBX3*, *ISL1*, and *NKX2.5*) (Figures S3B–S3D). Telomere repeat amplification protocol (TRAP) showed that silencing of *hTERT* mRNA is paralleled by decreased TERT catalytic activity at day 10 (Figures S3A and S3E). However, we observed residual telomerase activity even at day 10 of differentiation, which presumably arises from non-CMs in the mixed pool of differentiated cells. Hence, we implemented a differentiation protocol that included a CM selection step as well as a ∼60 day maturation period (Figure S3F). Following the 60 day protocol, *NANOG* expression decreased (Figure 5A), whereas the expression of *NKX2.5* increased (Figure 5B) compared to hiPSCs (day 0), as expected. A more mature phenotype of the hiPSC-CMs at day 60 was observed compared to day 25, as quantified by the ratio of *beta* and *alpha MHC* (Figure 5C). Immunostaining of the hiPSC-CMs at day 60 for cardiomyocyte markers such as sarcomeric alpha actinin confirmed maturity at the structural level (Figure 5D). The purified population of hiPSC-CMs at day 60 neither expressed *hTERT* mRNA nor showed detectable TERT catalytic activity (Figures 5E and 5F). Thus, these CMs provide a human *in vitro* model to study the potential cardioprotective mechanisms mediated by *TERT* therapy.

### *TERT* therapy ameliorates doxorubicin toxicity in human cardiomyocytes

**Figure 6:**
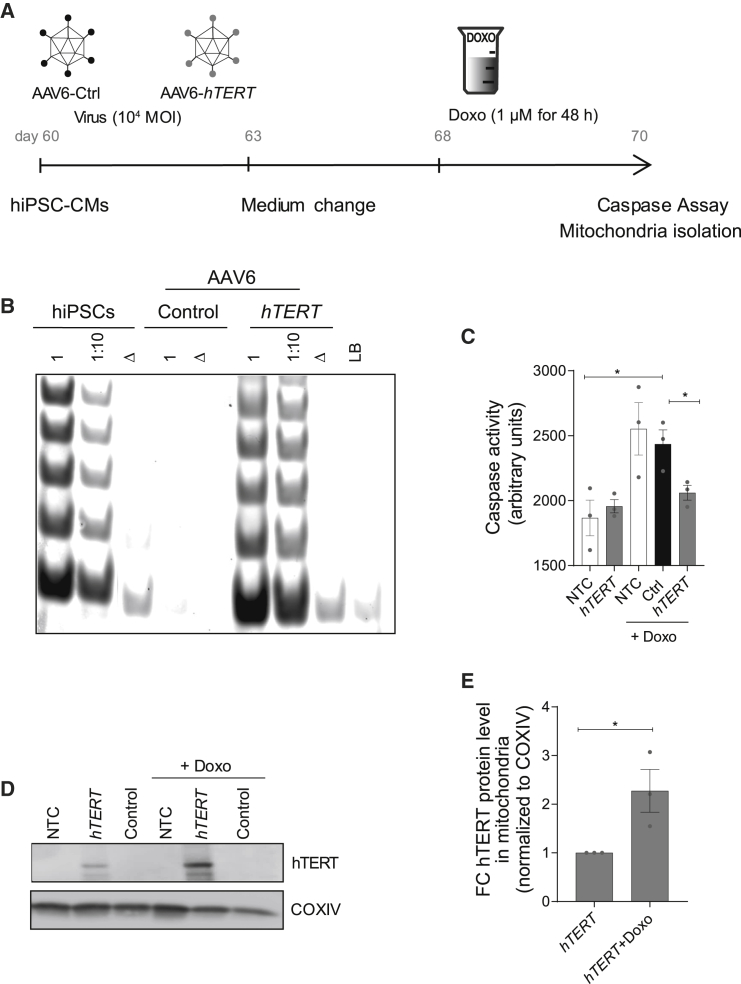
AAV6-*hTERT* inhibits doxorubicin-induced apoptosis via regulating mitochondrial dynamics (A) Schematic of experimental strategy for investigating cardioprotection post doxorubicin-induced stress. Overview of experimental time course for 7 day virus transduction (MOI 10^4^) and 48 h doxorubicin (1 μM) treatment in hiPSC-CMs. (B) Restoration of TRAP activity mediated via AAV6-*hTERT* transduction compared to AAV6 control. (C) Cell death measured by caspase activity before and after doxorubicin treatment in the presence and absence of AAV6-*hTERT* therapy indicated enhanced survival in the presence of *hTERT* overexpression. (D) Western blot indicating presence of TERT protein in the mitochondrial compartment, which further increases upon doxorubicin treatment. (E) Increase in hTERT protein levels within the mitochondria as measured from western blot. All data are mean relative to control ± SEM (n = 3 independent differentiation experiments). NTC, no virus treatment control; FC, fold change. ∗p < 0.05; ∗∗p < 0.01; ∗∗∗p < 0.001; two-tailed unpaired t test.

We again chose a viral delivery strategy to overexpress *hTERT* in the mature hiPSC-CMs at day 60. We first tested the efficiency of transduction by infecting hiPSC-CMs with AAV6-*EGFP* viral particles at an MOI of 10^4^. After 7 days, more than 80% of the hiPSC-CMs expressed EGFP, as analyzed by immunostaining (Figure S4A). Following this, AAV6-*hTERT* and AAV6-empty (AAV6-Ctrl) particles were produced to infect CMs again at a MOI of 1 × 10^4^. After virus transduction, the hiPSC-CMs were treated with doxorubicin for 48 h (Figure 6A). The AAV6-*hTERT* transduction resulted in a significant and stable induction of *hTERT* mRNA expression as well as TERT catalytic activity in 7 days (Figure 6B; Figure S4B). Doxorubicin strongly induced apoptosis in AAV6-Ctrl treated cells, whereas cells treated with AAV6-*hTERT* showed a significant rescue (Figure 6C).

### *TERT* translocates into mitochondria upon doxorubicin toxicity in human cardiomyocytes

In line with our *in vivo* data in mice, AAV6-*hTERT* did not alter the telomere lengths of largely post-mitotic hiPSC-CMs (Figure S4C). Prompted by our *in vivo* findings (Figure 4C), we next analyzed the alterations in mitochondrial morphology in hiPSC-CMs using transmission electron microscopy. In the setup of acute doxorubicin cardiotoxicity induced in hiPSC-CMs, we observed an increase in mitochondrial volume upon doxorubicin treatment, which was rescued by *hTERT* overexpression (Figures S4D–S4E). To further investigate a potential mitochondrial role of the TERT subunit, we isolated the mitochondria from hiPSC-CMs after doxorubicin treatment (Figure 6A). Strikingly, the amount of TERT protein translocating into mitochondria was significantly elevated under doxorubicin stress conditions (Figures 6D and 6E), further substantiating mitochondria-related functions of TERT.

### TERT protects mitochondrial damage induced by ROS and preserves mitochondrial function in human cardiomyocytes treated with doxorubicin

**Figure 7:**
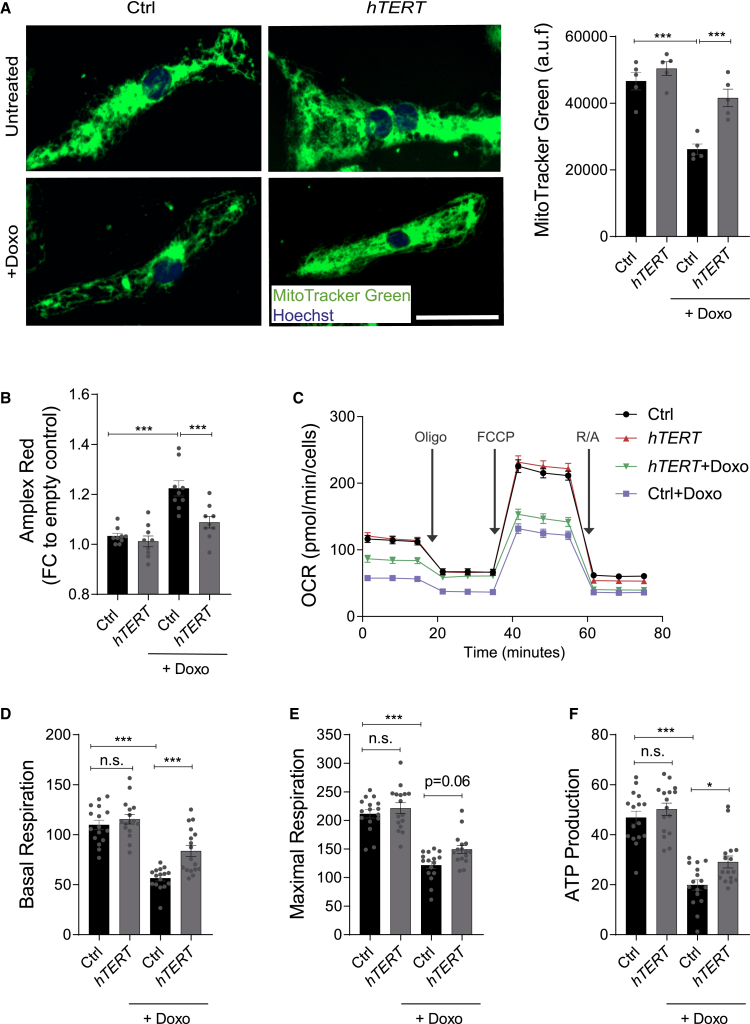
AAV6-*hTERT* protects from ROS and preserves mitochondrial metabolism post doxorubicin-induced cardiotoxicity (A) HiPSC-CMs overexpressing hTERT stained with MitoTracker Green FM in presence and absence of doxorubicin (1 μM, 48 h) and compared to control group. The fluorescence signal of MitoTracker Green FM from images on the left are quantified and represented on the right, which indicate mitochondrial content (n = 5 wells/group with 100,000 cells in each well from one differentiation experiment). Scale bar is 50 μm. (B) Bar graph represents the fold change of the extracellular ROS measured by Amplex Red assay before and after doxorubicin treatment in the presence and absence of AAV6-*hTERT.* The results indicate lower ROS levels after doxorubicin (1 μM) in the presence of *hTERT*. (n = 9 wells/group from triplicates of 3 independent differentiation experiments). (C) Analysis of hiPSC-CM mitochondrial metabolism using a Seahorse XFe96 Analyzer after transduction with AAV6 *hTERT* and in presence or absence of doxorubicin (1 μM). OCR was measured continuously at baseline and after addition of oligomycin (2 μM), FCCP (1 μM), and R/A (0.5 μM) (n = 16 wells/group with 50,000 cells in each well from one differentiation experiment). (D–F) The average levels of basal respiration, maximal respiration, and ATP production. The mitochondrial metabolism reduces after doxorubicin treatment, which is rescued by AAV6-*hTERT* therapy. All data are mean ± SEM. AAV6 transduction performed with 10^4^ MOI. OCR, oxygen consumption rate; oligo = oligomycin; FCCP, carbonyl cyanide-4-phenylhydrazone; R/A, rotenone and antimycin A. ∗p < 0.05; ∗∗∗p < 0.001; one-way ANOVA, Tukey multiple-comparisons test.

To gain further insights into the role of hTERT translocated within the mitochondria, we analyzed different aspects of mitochondrial dynamics and function. We performed live staining in hiPSC-CMs using MitoTracker Green FM, which covalently binds to mitochondrial proteins and represents an indirect measure of mitochondrial density.^19^^,^^20^ We observed a significant decrease in mitochondrial content of the hiPSC-CMs upon doxorubicin treatment, which was preserved in the presence of AAV6-*hTERT* therapy (Figure 7A). Doxorubicin toxicity is also attributed to the generation of ROS.^21^^,^^22^ In order to detect ROS production, we applied the Amplex Red reagent as it reacts with extracellular H_**2**_O_**2**_ in the presence of horseradish peroxidase, to produce highly fluorescent resorufin. Doxorubicin treatment in hiPSC-CMs resulted in very high levels of ROS generation, which were significantly restored in the cells receiving AAV6-*hTERT* therapy (Figure 7B). Finally, we measured the oxygen consumption rate (OCR) as a parameter of the mitochondrial function by using the seahorse analyzer system. Lower OCR measurements in doxorubicin-treated hiPSC-CMs were observed, which were rescued in the AAV6-*hTERT* group (Figure 7C). The rescue in mitochondrial function was further confirmed by higher rate of basal respiration, maximal mitochondrial respiration, and ATP production in the hTERT group compared to the control (Figures 7D–7F). The rates of proton leak and coupling efficiency exhibited better results in presence of hTERT overexpression, even though there was not a significant difference between basal and doxorubicin treatment (Figures S5A and S5B). However, the spare respiratory capacity and non-mitochondrial OCRs remained unchanged (Figures S5C and S5D).

Taken together, these results confirm that the TERT subunit localized within the mitochondria under doxorubicin stress helps to maintain the mitochondrial content, reduces ROS levels, and even restores mitochondrial function. Collectively, these protective effects in the mitochondria lead to enhanced survival of CMs under doxorubicin-induced cardiac stress.

## Discussion

The canonical role of telomerase is to compensate the loss of telomere repeats at the ends of eukaryotic chromosomes during cell division. However, in most somatic cells telomerase is silenced after birth, leading to gradual shortening of telomeres and eventually contributing to the onset of several age-associated disorders, including cardiovascular disease.^23^ One strategy to enhance telomerase activity is the use of sex hormones, which proved successful in preclinical models^24^ and in patients suffering from aplastic anemia due to haploinsufficient TERT expression.^25^^,^^26^ However, such potential activators of telomerase require a residual amount of endogenous telomerase activity, which is completely absent in adult CMs. Recent studies have highlighted protective effects of telomerase in terminally differentiated, non-dividing cells such as CMs. A previous study demonstrated that physical exercise in mice induces cardiac *Tert* expression concomitant with the activation of anti-apoptotic programs.^27^ Although the cell types that contributed to the increased *Tert* expression remained unidentified, the enhanced *Tert* expression promoted CM survival in a setting of acute doxorubicin-induced cardiotoxicity (single doxorubicin injection of 22.5 mg/kg). Interestingly, the beneficial cardiac effects of physical exercise were entirely absent both in *Tert* knockout mice and in mice deficient for nitric oxide synthase 3 (eNOS^−/−^)^27^. Since eNOS is specifically expressed in endothelial cells,^28^ this suggests that the protective effects observed in this study had presumably originated from the cardiac vasculature. While DNA intercalation and topoisomerase II poisoning are the main traits that account for the cytostatic effects of doxorubicin in cancer cells, these are insufficient to explain the toxicity in an extremely low turnover tissue such as the heart. Indeed, there is still no consensus on the precise molecular mechanism of doxorubicin-induced cardiotoxicity, but excessive production of ROS has emerged as the main contributor, as CMs are particularly prone to oxidative stress due to their high mitochondrial density.^11^ In this study, we investigated the cardioprotective traits of AAV-mediated telomerase overexpression specific to the cardiomyocyte cell population under doxorubicin stress conditions. We employed a small animal model and further translated our findings to the hiPSC-CMs in the context of doxorubicin-induced cardiotoxicity. Our study suggests that telomerase therapy could be a novel strategy for the prevention of anthracycline-induced cardiotoxicity via non-canonical telomerase functions in the mitochondria. AAV gene therapy led to high and stable gene expression of telomerase in the heart throughout the course of the experiment. The AAV particles were also able to provide robust increase of *hTERT* mRNA and TERT activity in hiPSC-CMs. Mice expressing telomerase exhibited preserved heart weight in comparison to controls (no telomerase expression) after chronic doxorubicin administration. Histopathological analyses revealed that this rescue was accompanied by preserved cardiomyocyte size and decreased apoptosis. This was also reflected by improved LV function. Complementary *in vitro* experiments confirmed anti-apoptotic effects of telomerase overexpression in doxorubicin-treated hiPSC-CMs.

However, these cardioprotective effects were not associated with altered telomere length, since treatment with doxorubicin or *Tert* (in mice) and *hTERT* (in hiPSC-CMs) did not cause telomere shortening or lengthening, respectively. Instead, we observed altered mitochondrial morphology and higher levels of mitochondrial fission proteins post doxorubicin-induced cardiotoxicity *in vivo*. The fusion process in mitochondria is also a key factor that modulates apoptotic signaling,^29^ highlighting the fact that mitochondrial fusion can act as a compensatory mechanism to enhance cell survival. In mice re-expressing telomerase, their ultrastructure analysis revealed the presence of a lower number of mitochondria, but the mitochondria were enlarged, pointing toward possibility of mitochondrial fusion, which also corroborated with the lower levels of mitochondrial fission proteins in these animals. Taken together, this might be a potential survival mechanism under doxorubicin stress conditions. Mitochondrial dynamics in response to doxorubicin cannot be recapitulated in CMs *in vitro*, since the rather acute doxorubicin treatment in hiPSC-CMs resulted in swelling of mitochondria, a phenomenon that has been described before.^30^ Nevertheless, our *in vitro* data showed that *TERT* treatment partially protected from this detrimental enlargement of mitochondria. Collectively, our data suggest that cardioprotective effects are at least partially exerted through mitochondrial mechanisms. This notion was further corroborated by demonstrating that upon doxorubicin cardiotoxicity, ectopically expressed TERT translocated into the mitochondrial compartment and further promoted CM cell survival. Recently there have been several reports on non-nuclear functions of telomerase under stress conditions that have highlighted its potential as a therapeutic target for ROS.^8^^,^^31^, ^32^, ^33^ Indeed, Beyer and colleagues^9^ showed that enforced expression of TERT in the mitochondria rescued endothelial cells from ROS-induced dysfunction, while pharmacological activation of the TERT subunit regulated mitochondrial ROS in endothelial cells of patients with CAD. Our data show that TERT localizes to the mitochondria in human CMs upon therapeutic overexpression and that it significantly accumulates in mitochondria under doxorubicin stress. Once localized to the mitochondria, TERT helps to maintain the mitochondrial mass, ROS levels, and metabolic functions under doxorubicin stress conditions. This strongly supports a non-canonical role of telomerase in the mitochondria that protects CMs from doxorubicin-mediated toxicity. In order to overcome the physiological limitations of the hiPSC-CM model, further studies in sophisticated multicellular or 3-dimensional models of hiPSC-CMs may provide further insights.^34^^,^^35^ Collectively, our data suggest that telomerase therapy could be a novel strategy for the prevention of chemotherapy-induced cardiotoxicity via non-canonical telomerase functions in the mitochondria by rescuing the heart from ROS-generated side effects of anthracyclines.

## Materials and methods

### Doxorubicin-induced cardiotoxicity animal model

The local animal authorities at Hannover Medical School and Niedersachsen Landesamt für Verbraucherschutz granted permission for all the animal experiments (licence number TVA 14/1665). A previously described mouse model of doxorubicin-induced cardiotoxicity was used.^15^ 12-week-old mice were subjected to AAV-based gene therapy (AAV9 1 × 10^12^ vg/mouse). AAV9-control or AAV9-*hTERT* particles were administered a week before starting the doxorubicin treatment. Mice in the sham group received AAV9-*Tert* virus together with saline injections. Adult C57BL/6N male mice were administered doxorubicin at a dose of 5 mg/kg weekly for 5 consecutive weeks to establish doxorubicin-induced cardiotoxicity (cumulative dose, 25 mg/kg bodyweight). We performed the *in vivo* experiments in male mice only, as literature strongly indicates that female rodents are resilient to doxorubicin-induced cardiotoxicity.^36^ Moreover, AAV-based gene therapy exhibits similar efficacy irrespective of sex of the animals.^37^ One week after the last injection, echocardiography and PV analysis via cardiac catheterization was performed to assess the cardiac function. Mice were then euthanized, and hearts were harvested for full histopathological and molecular analysis. Echocardiography was performed using Vevo 2100 system (FUJIFILM VisualSonics) under 2% isoflurane anesthesia. Mice were placed on the temperature-controlled Vevo mouse-handling table and connected to the anesthesia system via breathing mask. All paws were fixed on electrode plates to measure electrocardiogram (ECG). Warmed ultrasound gel was used on the thorax to establish acoustic connection with transducer. Measurements were done to evaluate cardiac and circulatory parameters in B- and M-mode, both in parasternal long- and short-axis views. During measurements, heart rate was kept around 460 ± 43 bpm. Images were acquired and analyzed with the standard imaging protocols (M-mode and B-mode) using Vevo LAB software 3.1.0 (FUJIFILM VisualSonics). PV measurements were acquired using a 1F microtip PV catheter (PVR 1045; Millar Instruments, Houston, TX, USA) coupled with a Powerlab/4SPacquisition system (AD Instruments, Oxford, UK) under constant maintenance of 37°C. PV loops were analyzed with Labchart 7 AD Instruments, Oxford, UK). All volume-based parameters were calibrated to the cardiac volumes acquired by echocardiography. All the animal experiments were randomized and blinded.

### TUNEL

TUNEL staining was performed per the manufacturer’s instructions, using an *in situ* cell death detection kit (Roche) as previously described.^15^ Heart cryosections were fixed with 4% paraformaldehyde (PFA) for 20 min at room temperature (RT) and then permeabilized with ice-cold 0.1% Triton X-100 in PBS for 2 min at RT. Cells or sections were incubated with the enzyme-labeling solution provided with the kit (Roche) for 1 h at 37°C. Cells were then incubated with 4′,6-Diamidin-2-phenylindol (DAPI) for 15 min. Three times washing was done at every step to remove residual substances. Images were acquired using the Nikon Eclipse Ti microscope and analyzed with NIS Elements.

### Cell size measurements

For measurement of cell size, paraffin-embedded heart sections were fixed and stained with Alexa Flour 488-labeled wheat germ agglutinin (WGA; Invitrogen) and DAPI. Images were taken using Nikon Eclipse Ti microscope and were analyzed with NIS Elements. Five to six different regions of the heart were imaged, and the area of 30 CMs was measured and averaged per animal. The cell surface area of CMs was calculated using the NIS-Elements BR 3.2 package (Nikon Instruments).

### Cloning and AAV production

The AAV-MCS vector (Cell Biolabs; #VPK-410) was cut open with BstBI enzyme and self-ligated to eliminate the beta-globin intron region to enhance the packaging limit of the virus. Further, the cTNT promoter was amplified from the pAAV:cTNT::Luciferase plasmid (Addgene; #69915) using primers that generated MluI and EcoRI overhangs and cloned into the AAV-MCS plasmid to generate the AAV-cTNT plasmid. The mTERT and hTERT sequence was sub-cloned using EcoRI or EcoRII/HindIII sites to generate the AAV-cTNT-mTERT and AAV-cTNT-hTERT constructs, respectively. The efficiency of the cTNT promoter has been characterized previously.^38^ AAV9 and AAV6 were produced as previously described.^15^ In short, the transgene AAV plasmid and the helper plasmid were transfected into HEK293T cells using polyethylenimine (Polysciences, 24765). After 96 h, the virus particles were harvested and purified from Benzonase-treated cell lysates over an iodixanol density gradient (OptiPrep, Fresenius Kabi Norge). AAV titers were determined by a standardized quantitative real-time PCR on vector genomes using the primers specific for CMV or cTNT sequence (Table S3). AAV9 was injected intravenously at a dose of 2 × 10^12^ viral particles. AAV6 was used at a MOI of 10^4^ for hiPSC-CMs.

### Western blot

Cell lysis and protein isolation and quantification were performed as described previously.^13^ Protein (50–30 μg) was run on an SDS gel and further transferred on a polyvinylidene fluoride (PVDF) membrane (Bio-Rad) via wet blotting. The respective antibodies and their dilutions were used as follows to detect the proteins: vinculin (1:5,000), Sigma-Aldrich #V9131; GAPDH (1:20,000), Abcam #ab8245; SOD2 (1:2,000), Abcam #ab16956; catalase (1:1,000), Cell Signaling Technologies #cs14097; pDRP1 (1:250), Sigma-Aldrich #SAB4301399; hTERT (1:100), Rockland #600-401-252S; COXIV (1:200), Cell Signaling Technologies #cs4844.

### RNA and quantitative real-time PCR

RNA was isolated using TriFast (Peqlab) as per the manufacturer’s instructions. Concentration of the isolated RNA was measured using Take3 Plates on a Bio-Tek plate reader (Synergy HT). For mRNA measurements, RNA (500–1,000 ng) was reverse transcribed using the iScript Select cDNA Synthesis Kit (Bio-Rad) or Biozym cDNA synthesis kit. A SYBR Green-based quantitative real-time PCR was performed with iQ SYBR Green mix (Bio-Rad). The quantitative real-time PCR was performed in ViiA7 (Applied Biosystems) machine using specific primer pairs (Table S3).

### hESC and hiPSC maintenance and differentiation

Culture and directed cardiomyogenic differentiation of the hESC line HES3 NKX2-5eGFP was performed as described previously.^39^^,^^40^ hiPSC line Phoenix (hHSC_Iso4_ADCF_SeV-iPS2, alternative name: MHHi001)^41^ and hCBiPSC2^42^ were cultured on Geltrex (Gibco #A1413302) coated cell culture plates in StemMACS full medium with supplements. The confluent hiPSCs were passaged every 5 days with Versene (Gibco #15040-066) in StemMACS (Miltenyi #130-104-368) full medium supplemented with 2 μM Thiazovivin (Selleckchem #S1459). HiPSC-derived CMs were differentiated and maintained as described previously^43^^,^^44^.

### Doxorubicin treatment in hiPSC-derived cardiomyocytes

Doxorubicin (Sigma-Aldrich) was applied at a dose of 1 μM for 48 h by adding into the hiPSC-CM maintenance medium (RPMI 1640 + GlutaMAX supplemented with 1× B27 with insulin; Thermo Fisher Scientific). All the *in vitro* experiments were performed as three biological replicates from three separate rounds of hiPSC-CM differentiation, unless otherwise mentioned.

### Caspase assay

Caspase assay was performed as per the manufacturer’s instructions with the Caspase-Glo 3/7 kit (Promega). Briefly, Phoenix hiPSC-CMs were treated with 1 μM doxorubicin in normal maintenance medium for 48 h. After 48 h, an equal amount of caspase assay reagent (pre-warmed at RT) was added and further incubated for 60 min at 37°C. Luminescence was measured using the HT Synergy (BioTek Instruments) plate reader.

### TRAP

TRAP assay was performed as described previously,^45^ with a few modifications. Briefly, aliquots of live cells were collected in nuclease-free 1.5 mL Eppendorf tubes via spinning down cell suspension at 3,000 × *g* for 5 min at RT. The supernatant was completely discarded, and the cell pellet was snap frozen in liquid nitrogen and stored at −80°C until processed further. The cell pellets were re-suspended in ice-cold NP-40 lysis buffer at a final concentration of 1,000 cells/μL and incubated on ice for 30 min to lyse the cells. Next, some of the cell lysate was used for heat inactivation at 85°C for 10 min and some for preparing a 1:10 dilution in nuclease-free water. The TRAP master mix was prepared using 50× primer mix (100 ng/μL each of ACX and NT primers along with 0.001 attomol/μL TSNT primers; see Table S4 for primer sequences). 2 μL of cell lysate was added to 48 μL of the master mix in nuclease-free tubes. Tubes were then placed in a thermocycler and run with the TRAP-PCR protocol (25°C for 30 min; 95°C for 5 min; 24 cycles of 95°C for 30 s, 52°C for 30 s, 72°C for 40 s). After the TRAP PCR, 20 μL of the PCR product for each sample was then loaded into wells of a 10% acrylamide electrophoresis gel (using 19:1 Acryl:Bis acrylamide in Tris-borate-EDTA [TBE] buffer) and resolved for 3 h at 4°C, shielded from light in a ProteanII xi 20 cm cell electrophoresis tank using the running buffer (0.5× TBE buffer). The gel was fixed using a fixative, then scanned using LICOR Odyssey scanner (#9120).

### Quantitative fluorescence *in situ* hybridization (qFISH)

To determine the telomere length in the heart from the various conditions, the paraffin-embedded tissue sections were processed and stained with telomere probe as described previously.^6^^,^^24^ Also, the telomere lengths were measured in hiPSC-CMs treated with and without AAV6 hTERT virus in presence and absence of doxorubicin stress. Briefly, 10,000–100,000 cells of hiPSC-CMs were spotted to Superfrost glass microscope slides using a Cytospin 3 centrifuge (Thermo Shandon) at 800 rpm for 8 min at RT. The slides were washed in 1× PBS (Ca^2+^/Mg^2+^ free) for 15 min in a shaker two times, followed by a fixation step of 4% formaldehyde (in 1× PBS) for 2 min. The slides were washed thrice in PBS for 5 min each. Slides were then treated with pre-heated acidified pepsin for 10 min in a 37°C water bath to digest the cells. The slides were washed twice with 1× PBS for 5 min each. Cells were fixed in 4% formaldehyde solution again for 2 min, followed by 1× PBS washes three times for a total of 15 min. Slides were then processed the same way as done with paraffin sections previously.^6^ Lastly, slides were air-dried before adding 10–15 μL of Antifade Vectashield mounting medium and sealed with coverslips using nail polish and stored in the dark at 4°C. Images of 40 nuclei per slide were acquired using Leica SP8 confocal microscope using 60× oil objective and additional 3× zoom with 405 nm and 561 nm lasers. Confocal images of qFISH performed in heart tissue were acquired using Zeiss LSM 780. Telomere qFISH in tissue sections was analyzed using Definiens Developer XD software. Telomere qFISH images of cultured cells were analyzed with ImageJ and a Telometer Plug-in^46^ (Created by Johns Hopkins University, NIH, USA). Telomere length is represented as arbitrary fluorescence units (a.f.u.).

### Isolation of mitochondria from hiPSC-CMs

Mitochondria were isolated from the cells using the mitochondria isolation kit, human (Miltenyi #130-094-532) as per manufacturer’s protocol. Phoenix hiPSC-CMs (∼5 million) were harvested and re-suspended in ice-cold PBS at a final dilution of 1 million cells/mL. The cells were lysed using 1 mL ice-cold lysis buffer (containing protease inhibitor) per 5–10 million cells by 20 strokes with the Dounce homogenizer. The cell lysate was transferred into a 15 mL falcon, diluted with 1× separation buffer, and further incubated with anti-TOM22 microbeads for 1 h at 4°C on a rotator to ensure magnetic labeling of the mitochondria within the lysate. After 1 h incubation, the mixture was passed through the pre-washed MACS LS column (#130-042-401), which ensured that magnetically labeled mitochondria would stick along the MACS column and all other non-mitochondrial population would get washed away with subsequent washing steps. In the last step, 1.5 mL of 1× separation buffer was added onto the column, and the mitochondrial fraction was collected in a fresh 15 mL falcon by pushing the plunger into the column. The collected mitochondrial suspension was transferred into a 1.5 mL Eppendorf and centrifuged for 2 min at 13,000 × *g* at 4°C. The supernatant was discarded, and the pellet was immediately processed for protein measurement or stored at −80°C.

### Immunostaining

Purified Phoenix hiPSC-CMs were seeded into a 96-well cell culture plate and given minimum 3 days to acclimatize. Cells were fixed with 4% formaldehyde, RT, and washed thrice with 1× PBS for 5 min each. Ice-cold 0.1% Triton X-100 was added for permeabilization at RT for 30 min. This was followed by three more washing steps with 1× PBS. Next, 5% donkey serum was applied as a blocking reagent and incubated for 1 h at RT. The primary antibody (sarcomeric alpha actinin [1:300, Sigma; A7811] or cardiac troponin T [1:500, Abcam; ab8295] or GFP [1:1000, Abcam; ab6556]) was prepared in the blocking buffer, and cells were incubated at 4°C overnight. The next day, after three washing steps, the appropriate secondary antibody (donkey anti-mouse Alexa Fluor 546 red, Invitrogen; A10036 or donkey anti-mouse Alexa Fluor 594 red, Invitrogen; A21203 or donkey anti-rabbit Alexa Fluor 488, Invitrogen; A21206) dilution (1:500) was added to the cells and incubated in the dark at RT for 2 h. The wells were washed twice for 5 min each in 1× PBS. Further, 1:1,000 solution of Hoechst 33342 (1:1000, Thermo Fisher; #62249) in 1× PBS was applied for 5 min at RT in the dark. Each well was washed with 1× PBS once more for 5 min, and finally fresh PBS was added before storing the plate until microscopic analysis.

### Electron microscopy

Small pieces of explanted heart tissues were immersion-fixed in 150 mM HEPES (pH 7.35) containing 1.5% formaldehyde and 1.5% glutaraldehyde (GA). Tissue embedding and heart section preparation were done at the Institute of Functional and Applied Anatomy, Hannover Medical School (MHH) as previously described.^47^ The Phoenix hiPSC-CM inserts were fixed in fixative solution composed of 1.5% PFA, 1.5% GA, and 0.15 M HEPES (pH 7.35) and processed further as described previously.^48^ Several grids were prepared from different regions of the insert. The entire grid (from three different regions of one insert/condition) was imaged to sample in an unbiased approach. Electron microscopic examinations were performed at the Hannover Medical School, electron microscopy facility, with the FEI Morgagni 268 transmission electron microscope (FEI, Eindhoven, the Netherlands) operated at 80 kV using a Veleta CCD camera (Olympus Soft Imaging Solutions).

### Mitochondrial volume analysis of electron microscopic images

Images acquired using the electron microscope were analyzed in an unbiased approach with the help of the newCast software (Visiopharm, Hørsholm, Denmark). A virtual overlay with a line grid with 20 points and a length per point of 0.85 μm was applied on the individual images. All the lengths along the test line were measured in each mitochondrion that was sampled by the point grid with the point sampled intercept (PSI) method. With this unbiased scoring method, the density of mitochondria and their respective lengths (l) were estimated. The calculation of the volume-weighed mean mitochondrial volume (v_V_[mito]) was carried out with the following formula:^49^

$$\;{\text{v}}_{\text{V}}\left(\text{mito}\right)\;=\text{πx }{\left(\text{mean l}\right)}^{3}/3\;$$

### Amplex Red assay

Extracellular H_2_O_2_ released by cells was determined using 10-acetyl-3,7-dihydroxyphenoxazine (Amplex Red reagent) using the reagents and protocols provided in the Amplex Red Hydrogen Peroxide Assay Kit (Molecular Probes; #A22188). Phoenix hiPSC-CMs were seeded on 48-well culture plates at a density of 100,000 cells per well. The hiPSC-CMs were transduced with AAV6 virus expressing hTERT and, as control, the empty virus in presence and absence of 1 μM doxorubicin stress in normal maintenance medium for 48 h. The cells were washed with the PBS. A working solution of the Amplex Red reaction mixture (10 μM Amplex Red and 0.2 U/mL horseradish peroxidase) in PBS (pH 7.4) was prepared, and 250 μL was added to each well. This was followed by a 30 min incubation period at 37°C. The cells were washed once with the PBS. Amplex Red conversion to resorufin was measured at emission of 595 nm (excitation 535 nm) using the HT Synergy (BioTek) plate reader.

### MitoTracker Green FM staining and quantification

The mitochondrial density was measured using MitoTracker probes (Cell Signaling Technologies; #9074P). This probe is cell permeable and contains a mildly thiol-reactive chloromethyl moiety for mitochondrial labeling. Phoenix hiPSC-CMs were seeded on 48-well culture plates at a density of 100,000 cells per well. The hiPSC-CMs were transduced with AAV6 virus expressing hTERT and, as control, the empty virus in presence and absence of 1 μM doxorubicin stress in normal maintenance medium for 48 h. MitoTracker Green FM (500 nM) and Hoechst 33342 (1:1,000, Thermo Fisher; #62249) working solution was prepared in Opti-MEM reduced serum media (Gibco; #51985-026) and loaded on the live cells for 15 min at 37°C. The cells were washed once with Opti-MEM reduced serum media. MitoTracker Green FM fluorescence intensity was measured at emission of 530 nm (excitation 485 nm). Each group consisted of 5 wells, and the fluorescence intensity of the entire well with 100,000 cells was measured. The fluorescence intensity and images were acquired using Cytation 1 (Biotek).

### Seahorse extracellular metabolic flux assay

Phoenix hiPSC-CMs were plated at a density of 50,000 cells per well in XFe96 cell culture microplates coated with Matrigel. The hiPSC-CMs were transduced with AAV6 virus expressing hTERT and, as control, the empty virus in presence and absence of 1 μM doxorubicin stress in normal maintenance medium for 48 h. The XF Cell Mito Stress Test Kit was used as per the manufacturer’s protocol to study the mitochondrial function of hiPSC-CMs under basal and doxorubicin stress conditions. The bioenergetics were measured with the Seahorse XFe96 Analyzer. Media was aspirated and cells were washed twice and finally replaced with 180 μL assay medium (Agilent; #103575-100) and pre-equilibrated for 1 h at 37°C. Baseline OCR measurements were acquired, followed by injection of 2 μM oligomycin, 1 μM carbonyl cyanide-4-phenylhydrazone (FCCP), and finally 0.5 μM rotenone and antimycin A. Each injection was followed by three OCR measurements. The seahorse-run hiPSC-CMs were stained with Hoechst 33342 (1:1,000, Thermo Fisher; #62249) and imaged using Cytation 1 (Biotek). The cell numbers were used to normalize the seahorse data, and 16 wells per group were analyzed in this metabolic assay.

### Statistics

All the data were analyzed with GraphPad Prism 8 and are presented as mean ± SEM. An unpaired two-tailed t test was conducted to compare two groups wherever required. For comparison of ≥3 groups, a one-way ANOVA was conducted with a post hoc Tukey test or a Dunnett’s multiple comparison test. In case of significant difference among SDs, a one-way ANOVA with Welch’s correction was conducted to compare two groups.

## Supplemental information

Document S1. Figures S1–S5 and Tables S1–S4

Document S2. Article plus supplemental information
