# Supplementary material for: Telomerase therapy attenuates cardiotoxic effects of doxorubicin
Source: Mol Ther. 2021 Jan 1;29(4):1395–410. doi: 10.1016/j.ymthe.2020.12.035 (PMC8058493; doi:10.1016/j.ymthe.2020.12.035)
Supplement: Document S1. Figures S1–S5 and Tables S1–S4 [file mmc1.pdf]

YMTHE, Volume *29*

Supplemental Information

Telomerase therapy attenuates cardiotoxic effects of doxorubicin

Shambhabi Chatterjee, Teresa Hofer, Alessia Costa, Dongchao Lu, Sandor Batkai, Shashi Kumar Gupta, Emiliano Bolesani, Robert Zweigerdt, Diego Megias, Katrin Streckfuss-Bömeke, Christina Brandenberger, Thomas Thum, and Christian Bär

Figure S1

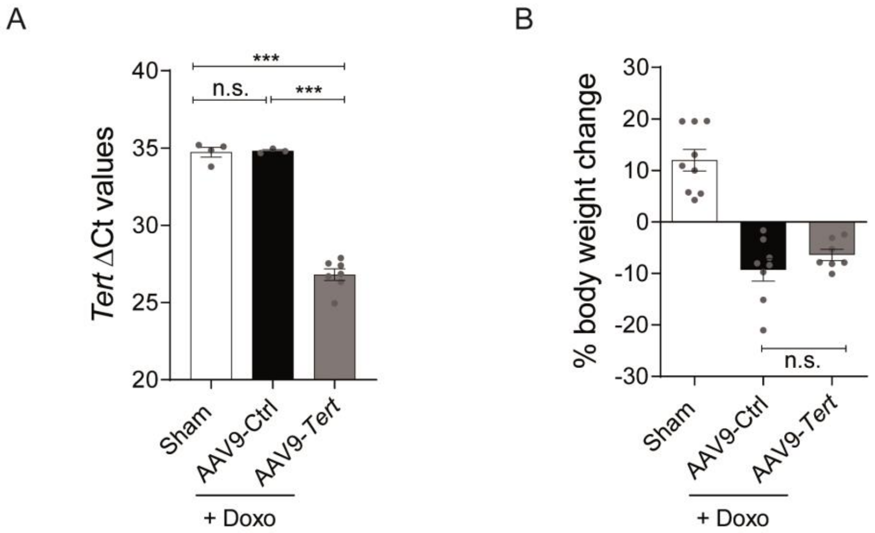

Figure S1| AAV9-*Tert* protects from doxorubicin-induced cardiotoxicity (A) Overexpression of *Tert* was confirmed by qPCR. The threshold ct values were lower in mice injected with AAV9-*Tert* particles indicating towards higher expression (n=4/6/7 mice). Animals with undetectable *Tert* mRNA expression (ct values above 36) were excluded from this graph. (B) Doxorubicin led to loss of body weight in mice which was partially rescued in the telomerase therapy group (n=9/8/7 mice). All data are shown as mean ± SEM). ***p<0.001; n.s.= not significant; One-way ANOVA, Tukey multiple-comparisons test.

Figure S2

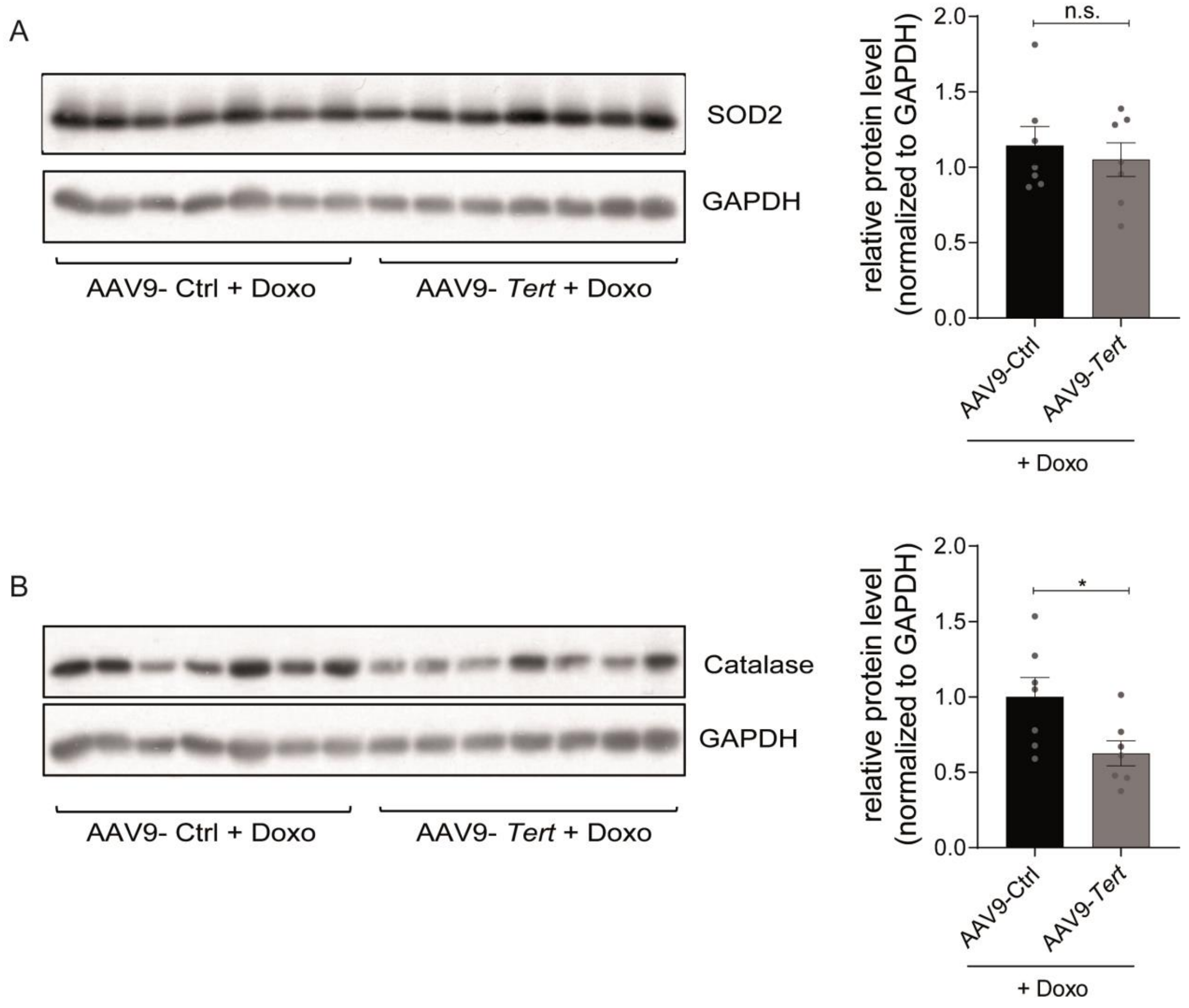

Figure S2| AAV9-*Tert* influences mitochondrial function and dynamics (A) Protein levels of SOD2 remained unchanged amongst the mice despite of *Tert* overexpression. (B) Mice injected with AAV9-*Tert* showed lower levels of catalase protein. All data are mean fold change relative to control ± SEM. (n=7 mice/group). Doxo= doxorubicin; *$p<0.05$, n.s.= not significant; Two-tailed unpaired t-test.

Figure S3

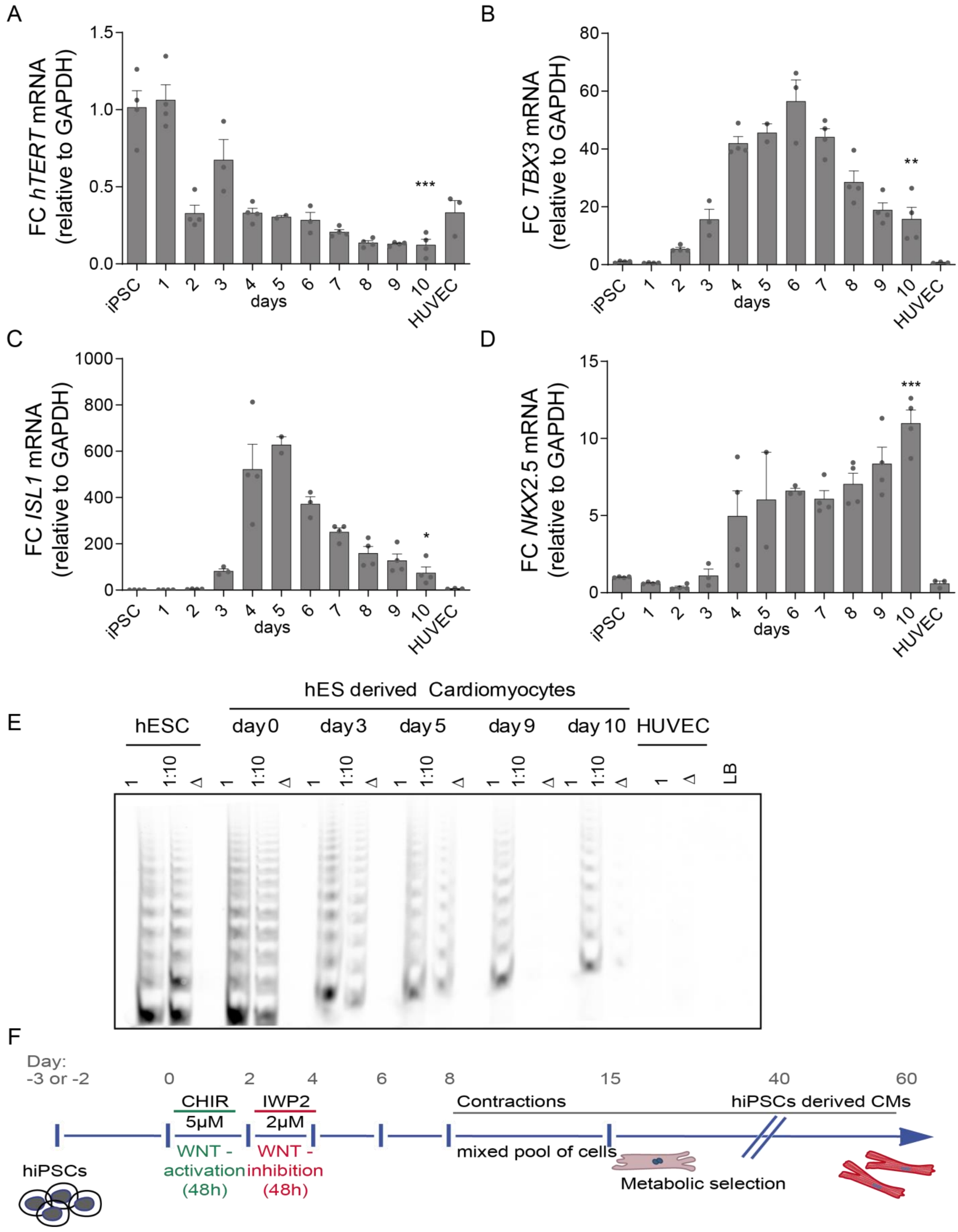

Figure S3| Validation of hiPSC-CMs as a translational platform for telomerase therapy (A) Expression of endogenous *hTERT* expression during differentiation. HUVEC used as a negative control with lower TERT expression. (B-D) mRNA expression of differentiation markers (*TBX3, ISL1, NKX2.5*) during the differentiation timeline (n=2-4 wells /group from one round of differentiation) day 10 time-point is compared to iPSCs for each marker. HUVEC used as a negative control for expression of differentiation markers. (E) Telomerase activity

during the first 10 days of the differentiation protocol as analyzed by TRAP assay. HUVEC used as a negative control with undetectable telomerase activity (F) Schematic representation of hiPSC-CM differentiation protocol with a metabolic selection procedure and prolonged culture maintenance. Δ indicates heat inactivated cell lysate; 1 indicates undiluted cell lysate; 1:10 indicates cell lysate diluted by factor 10; FC= fold change; All data are mean ± SEM. *$p<0.05$; **$p<0.01$; ***$p<0.001$; One-way ANOVA with Dunnett's multiple comparison test.

Figure S4

A

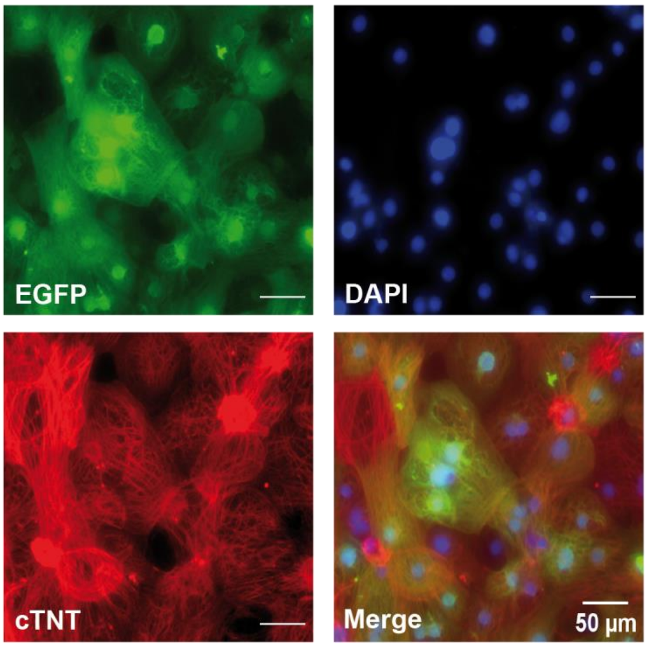

B

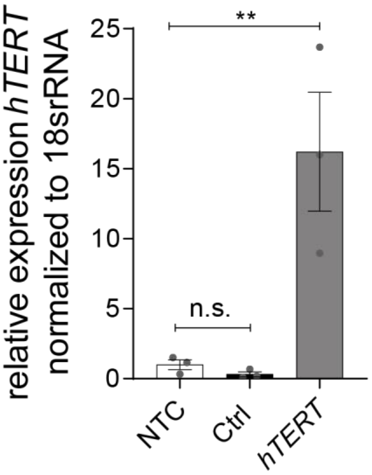

C

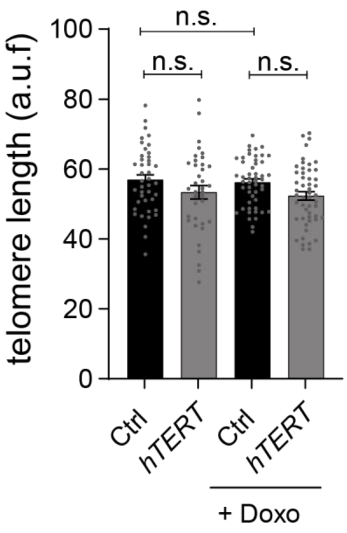

D

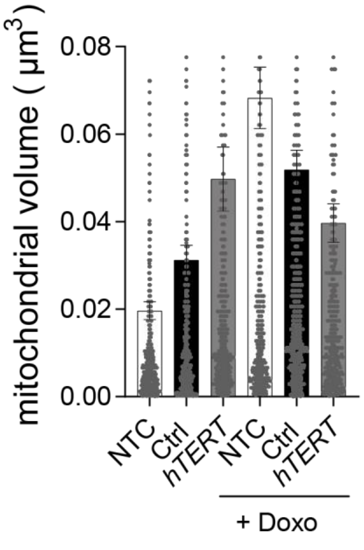

E

	Median Value
NTC	0.0066
Empty	0.0096
hTERT	0.0135
NTC+Doxo	0.0173
Empty+Doxo	0.0206
hTERT+Doxo	0.0135

Figure S4| AAV6-mediated *hTERT* overexpression in hiPSC-CMs does not alter telomere length but protects mitochondria under doxorubicin stress (A) HiPSC-CMs transduced with AAV6-*EGFP* viral particles at an MOI of 10^4. After 7 days of transduction more than 80% of the cells express EGFP protein. (B) Validation of *hTERT* mRNA expression after virus transduction with AAV6-*hTERT*. (n=3 independent differentiation experiments/group) (C) The telomere length measured in hiPSC-CMs using qFISH in presence and absence of telomerase therapy and with or without doxorubicin stress (n=37-52 nuclei/group) (D-E) Median mitochondrial volume quantified from hiPSC-CMs after telomerase therapy (n= a minimum of 425 mitochondria analyzed from 3 different regions of transmission electron microscopy samples). Scale bar indicates 50 µm. All data are mean ± SEM. Doxo= doxorubicin; NTC= no virus treatment control; a.u.f = arbitrary unit of fluorescence; *p<0.05; **p<0.01; ***p<0.001; One-way ANOVA, Dunnett's multiple comparison test.

Figure S5

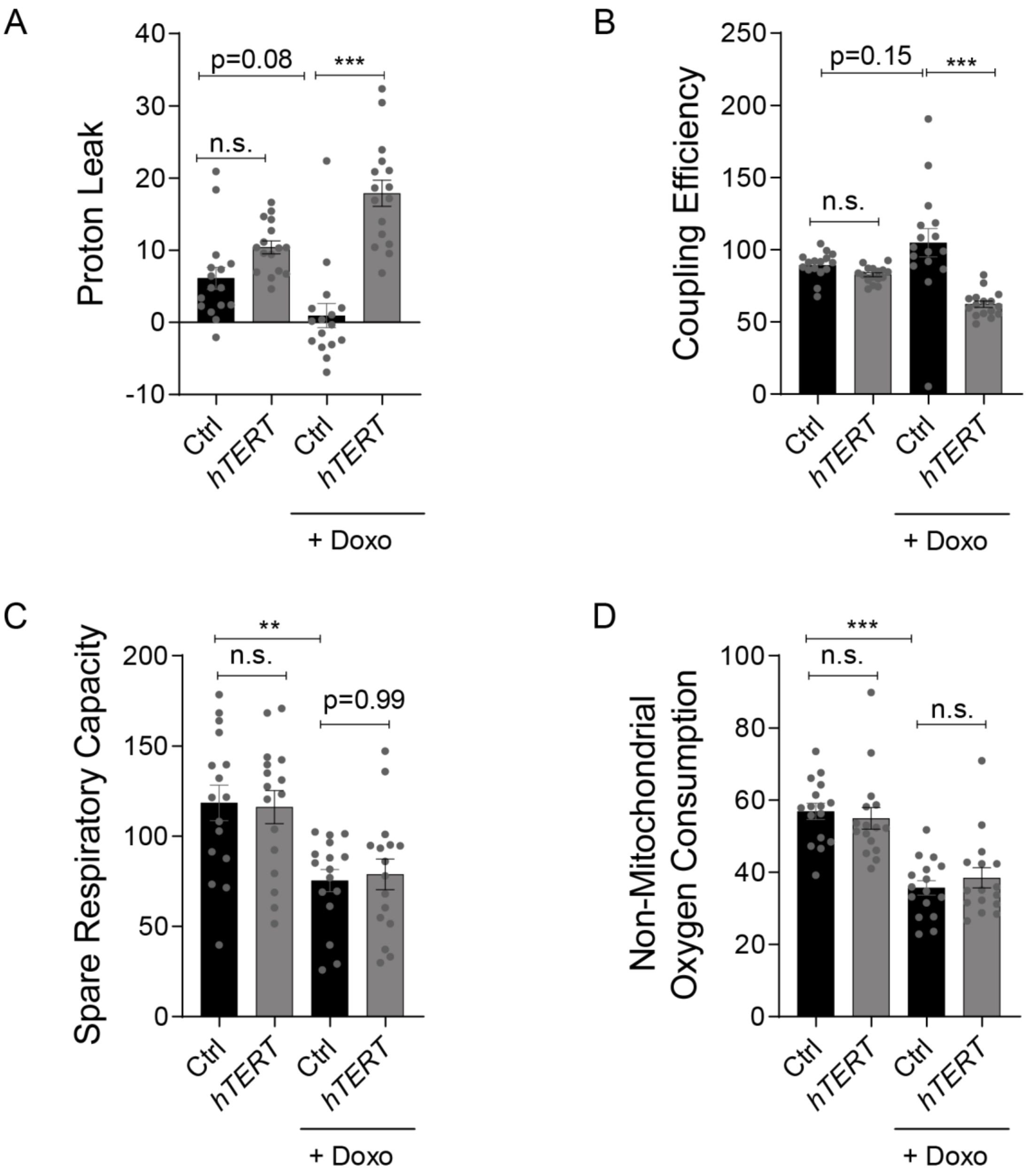

Figure S5| AAV6-*hTERT* therapy in hiPSC-CMs protects mitochondrial function under doxorubicin stress (A-D) Average levels of proton leak, coupling efficiency, spare respiratory capacity and non-mitochondrial oxygen consumption as measured by the seahorse analyzer (n=16 wells/group from one differentiation experiment). Proton leak is rescued by TERT therapy along with decrease in coupling efficiency post doxorubicin treatment. There is no significant change in the spare respiratory capacity and non-mitochondrial oxygen consumption in presence of AAV6-*hTERT* after doxorubicin treatment. All data are mean ± SEM. Doxo= doxorubicin; * p<0.05; *** p<0.001, n.s. not significant; One-way ANOVA, Tukey multiple-comparisons test.

Supplemental Table 1: List of Echocardiographic parameters

	**Sham**			**AAV9-Ctrl + Doxo**			**AAV9-*Tert* + Doxo**		
	Mean	SEM	N	Mean	SEM	N	Mean	SEM	N
HR	437.03	8.52	9	440.65	12.49	8	421.65	13.01	7
Diameter;s	2.87	0.20	9	2.80	0.12	8	2.65	0.07	7
Diameter;d	4.25	0.14	9	4.05	0.07	8	4.01	0.06	7
Volume;s	33.70	5.3	9	30.41	3.62	8	26.09	1.83	7
Volume;d	82.29	6.33	9	72.81	3.28	8	70.93	2.65	7
Stroke Volume	48.59	2.45	9	42.39	3.62	8	44.84	2.88	7
EF	60.94	3.97	9	58.29	4.06	8	62.96	2.62	7
FS	32.96	2.78	9	30.90	2.74	8	33.83	1.94	7
CO	21.20	1.08	9	18.91	2.00	8	18.41	2.02	7
LV Mass	155.17	5.61	9	116.32	7.59	8	123.56	10.31	7
LVMC	124.14	4.48	9	93.06	6.07	8	98.97	8.15	7
LVPW;s	0.87	0.05	9	0.63	0.03	8	0.89	0.09	7
LVPW;d	1.13	0.08	9	0.87	0.07	8	1.19	0.06	7

HR- Heart rate, EF- Ejection Fraction, FS- Fractional Shortening, CO- Cardiac Output, LV- Left ventricular, LVMC- Left ventricular Mass Corrected, LVPW- left ventricular posterior wall at end diastole (d) or systole (s)

Supplemental Table 2: List of invasive hemodynamic parameters

	**Sham**			**AAV9-Ctrl + Doxo**			**AAV9-*Tert* + Doxo**		
	Mean	SEM	N	Mean	SEM	N	Mean	SEM	N
SW (mmHg*µL)	2325.13	298.58	8	1716.37	210.19	8	1815.04	316.50	7
CO (µL/min)	12946.75	1594.07	8	12763.62	1488.76	8	11020.42	1606.75	7
SV (µL)	28.58	3.67	8	27.00	2.92	8	24.70	4.00	7
Pes (mmHg)	101.06	3.57	8	76.44	3.74	8	90.65	2.42	7
Ped (mmHg)	4.78	1.28	8	4.87	0.81	8	3.81	0.49	7
HR (bpm)	459.82	19.21	8	466.82	15.29	8	455.04	15.04	7
dP/dt max (mmHg/s)	9189	367.94	8	6860.5	798.91	8	9400	512.07	7
dP/dt min (mmHg/s)	-9798.75	424.45	8	-7394.62	809.73	8	-8495.71	232.99	7
Tau (ms)	6.97	0.27	8	8.22	1.44	8	7.5	0.43	7

SW-Stroke Work, CO- Cardiac Output, SV- Stroke Volume, Pes- End-systolic Pressure, Ped- End-Diastolic Pressure, HR- Heart rate, dP/dt max- Point of maximum pressure increase, dP/dt min- Point of maximum pressure decrease, Tau- time constant of active relaxation

Supplemental Table 3: List of primers used for mRNA quantification

**Gene name**	**Species**	**Primer sequence (5'->3')**
Beta-Actin	Mouse	Forward : CTGAGGAGCACCCTGTGCTG<br>Reverse : CCAGAGGCATACAGGGACAA
Tert	Mouse	Forward : GGATTGCCACTGGCTCCG<br>Reverse : TGCCTGACCTCCTCTTGTGAC
18s	Human	Forward : AGTCCCTGCCCTTTGTACACA<br>Reverse : GATCCGAGGGCCTCACTAAA
GAPDH	Human	Forward : CCAGGCGCCCAATACG<br>Reverse : CCACATCGCTCAGACACCAT
NANOG	Human	Forward : AGTCCCAAAGGCAAACAACCCACTTC<br>Reverse : TGCTGGAGGCTGAGGTATTTCTGTCTC
NKX2.5	Human	Forward : CAAGTGTGCGTCTGCCTTT<br>Reverse : CAGCTCTTTCTTTTCGGCTCTA
ISL1	Human	Forward : GCGGAGTGTAATCAGTATTTGGA<br>Reverse : GCATTTGATCCCGTACAACCT
TBX3	Human	Forward : CCCGGTTCCACATTGTAAGAG<br>Reverse : GTATGCAGTCACAGCGATGAAT
α- MHC	Human	Forward : GTGGACAAGCTGCAACTGAA<br>Reverse : GTCACTCCTCATCGTGCATT
β- MHC	Human	Forward : CCTGCTCTGTGTCTTTCCCT<br>Reverse : ACTGCCATCTCCGAATCTCC
TBP	Human	Forward : CCACTCACAGACTCTCACAAC<br>Reverse : CTGCGGTACAATCCCAGAACT
hTERT	Human	Forward : GCCTTCAAGAGCCACGTC<br>Reverse : CCACGAACTGTCGCATGT
CMV	AAV Titer	Forward : GCGTGGATAGCGGTTTGACT<br>Reverse : GGGCGGAGTTGTTACGACAT
cTNT	AAV Titer	Forward : AAGCTGCAGAAGTTGGTCGT<br>Reverse : TGGAGAGAAAGGCAAAGTGG

Supplemental Table 4: List of primers used in TRAP Assay

**Primer**	**Sequence (5'->3')**
TSNT	AATCCGTCGAGCAGAGTTAAAAGGCCGAGAAGCGAT
NT	ATCGCTTCTCGGCCTTTT
ACX	GCGCGGCTAACCCTAACCCTAACC
TS(DY-682)	AATCCGTCGAGCAGAGTT
